# Virtual Training of Endurance Cycling – A Summary of Strengths, Weaknesses, Opportunities and Threats

**DOI:** 10.3389/fspor.2021.631101

**Published:** 2021-03-04

**Authors:** Benjamin McIlroy, Louis Passfield, Hans-Christer Holmberg, Billy Sperlich

**Affiliations:** ^1^Department of Sport Science, Integrative and Experimental Exercise Science, University of Würzburg, Würzburg, Germany; ^2^Department of Sport and Public Services, Brooklands College, Weybridge, United Kingdom; ^3^Faculty of Kinesiology, University of Calgary, Calgary, AB, Canada; ^4^School of Sport and Exercise Sciences, University of Kent, Canterbury, United Kingdom; ^5^Department of Physiology and Pharmacology, Biomedicum C5, Karolinska Institute, Stockholm, Sweden; ^6^Department of Engineering Sciences and Mathematics, Luleå University of Technology, Luleå, Sweden

**Keywords:** algorithms, cycling, e-coach, e-health, ergometer, simulation, virtual training, SWOT

## Abstract

Virtual online training has emerged as one of the top 20 worldwide fitness trends for 2021 and continues to develop rapidly. Although this allows the cycling community to engage in virtual training and competition, critical evaluation of virtual training platforms is limited. Here, we discuss the strengths, weaknesses, opportunities and threats associated with virtual training technology and cycling in an attempt to enhance awareness of such aspects. Strengths include immersive worlds, innovative drafting mechanics, and versatility. Weaknesses include questionable data accuracy, inadequate strength and reliability of power-speed algorithms. Opportunities exist for expanding strategic partnerships with major cycling races, brands, and sponsors and improving user experience with the addition of video capture and “e-coaching.” Threats are present in the form of cheating during competition, and a lack of uptake and acceptance by a broader community.

## Introduction

In a recent survey, virtual and online training ranked in the top 6 worldwide fitness trends for 2021 (Thompson, 2021). Development of strategic digital live-streaming or pre-recorded sessions of group, individual, or instructional programs allows exercise to be performed at home (Thompson, 2021). This is particularly important at this time, since the national or local lockdowns used to manage the COVID-19 pandemic in many parts of the world, including temporary closure of gyms, has forced many athletes to engage in ergometer training at home.

In the case of cycling, advances in technology have improved indoor training equipment, providing novel simulation trainers equipped with power measuring capability connected online with new 2- or 3D virtual training and competition applications. The virtual environment can be achieved with wearable technology, such as a virtual reality (VR) headset, or through a figure on the screen (normally referred to as an avatar) whose movements the player controls. The most important feature of VR is effective immersion, making the individual feel fully present in the virtual environment (Witmer and Singer, 1998; Radianti et al., 2020).

In the rapidly evolving field of virtual online training, Zwift (https://www.zwift.com) is currently one of the most popular platforms, with more than 2.5 million registered app users in 190 countries (Long, 2020) and an all-time high of more than 30,000 users cycling at the same time (Schlange, 2020b). Other communities such as Peloton (https://www.onepeloton.com/), Real Grand Tours (RGT; https://www.rgtcycling.com/), Rouzy (https://rouvy.com/), and others are growing. The Zwift community continues to expand, with the formation of racing teams, first informally and more recently with support from sponsors, and competition monitored through a third party (Zwiftpower; https://zwiftpower.com/).

Here, we would like to share our experience concerning cycling in virtual reality by elite and amateur athletes worldwide. In this context we summarize the strengths, weaknesses, as well as opportunities and threats of virtual online training platforms (i.e., especially, but not exclusively Zwift) in attempt to enhance awareness of various aspects of virtual training technology and online cycling. This description is also intended to act as a starting point for discussion and planning of future research on this new and rapidly evolving type of sport.

## Strengths

### Availability at a Wide Range of Costs

With the Zwift online training platform, for example, power, speed, cadence, and heart rate are monitored indoors by specific sensors on a bicycle set up as a static trainer. Most popular are turbo trainers, free-standing rollers, or specially designed indoor bicycles. Turbo trainers vary in their level of technology, starting with wheel-on trainers (i.e., attaching the rear bicycle wheel to a weighted fly wheel), which are usually unpowered and unable to provide data, requiring external devices for this purpose (and therefore nicknamed “dumb” trainers). Top-of-the-line (“smart”) trainers are direct-drive (requiring the rear bicycle wheel to be removed and the bicycle chain to be attached directly to the trainer) and are usually electronic and capable of simulating conditions such as incline and changes in the road surface, while monitoring performance data.

To date, a wheel-on trainer plus external speed and cadence monitors costs ~£150/€165/$195 (not including the bicycle) in addition to the monthly Zwift subscription fee of £12/€13/$15. The cost can rise to more than £2,000/€2,200/$2,500 when purchasing a high-quality trainer, with incline simulation and a secondary power-meter for back-up and verification. Although the latter may seem to be relatively expensive, it is cheaper than other commercially available virtual training platforms utilizing VR headsets (Duking et al., 2018), as well as many of the cycle training camps organized in different parts of the world. This high-end set-up can provide an almost unlimited number of simulated training environments, routes, and races.

### Novel Strategies for Team Management

The adaptable nature of virtual cycling platforms allows preparation for many different kinds of competitions, decreasing the need for athletes and coaches to travel to different training venues, thereby avoiding jetlag and fatigue (Fowler et al., 2017), reducing the time lost to periodization and tapering, and costs normally associated with travel (Le Meur et al., 2012). In addition, coaching staff can assist athletes remotely, regardless of location or time zones. Cyclists can train and compete in greater comfort in their own homes and/or other familiar surroundings. Moreover, fewer mechanics will be required as the risk of problems with an indoor trainer is relatively low.

### Realistic Simulation of Many Different Racing Situations and Conditions

New routes and training environments are being developed continuously, with the most recent updates encompassing simulations of different stages of the Tour de France, including the world-famous sprint finish along the Champs-Élysées. Currently, more than 70 racing courses are available, ranging from short sprints (<5 km) to endurance courses (longer than 100 km). World-famous climbs, such as the Alpe d'Huez and Mont Ventoux, can be simulated in virtual reality. This versatility allows greater training specificity than is possible with more traditional indoor cycling.

Furthermore, virtual cycling platforms can simulate drafting effects that mimic those experienced outdoors, i.e., a cyclist is able to conserve energy by riding behind another cyclist on-screen. This drafting effect allows for basic adaptations based on differences in the cyclist's height and mass, the weight and model of the bicycle and wheel selection (in the Zwift simulation), the size of riding group, and inclines, even shallow climbs of up to 3 degrees. When cycling downhill, the rider can free-wheel and still maintain speed while in different positions, most notably the “super-tuck” position, an extremely aerodynamic position which every avatar will assume when free-wheeling at or above a certain speed. During actual cycling outdoors on varying terrain, freewheeling is quite common and, accordingly, virtual training platforms can simulate a range of cadences.

In addition, virtual cycling platforms allow simulation of different road surfaces, including tarmac, gravel, and dirt, each with its own resistance and riding experience. Thus, with only one type of bicycle at home, the athlete can train and compete in a greater variety of scenarios or categories than would otherwise be possible.

The most recent innovation is the introduction of steering capabilities via a steering platform fixed to the bicycle at home, which allows for an even greater level of immersion in the virtual environment.

### Safety

Globally, rates of traffic-related cycling injuries vary from 174 to 1,329 per 100,000 registered cyclists (Ag, 2019), resulting in significant costs – in the case of minor injuries, averaging 841 € in time lost from work, medical treatment, and costs for replacement of equipment (Aertsens et al., 2010). In addition, fear of, e.g., heavy traffic, darkness and/or bad weather, being attacked by strangers and bicycle theft is often a barrier to engaging in cycling (Heesch et al., 2012).

The ability to participate in simulated races in different disciplines and in large group races without fear of accidents is particularly useful for those recovering from an injury or who are anxious when cycling in groups. Nervous and inexperienced cyclists can also join a race on virtual cycling platforms without having to deal with the potentially intimidating experience of traveling to an outdoor event and negotiating the start of a mass participation event.

Athletes can also conduct high-intensity training sessions without encountering traffic or, e.g., having to stop at traffic lights, allowing training loads to be standardized. In fact, the sense of pressure and urgency that can be created in connection with virtual cycling can increase both the intensity and enjoyment of high-intensity interval cycling by untrained individuals (Farrow et al., 2019).

An additional advantage is that the cyclist does not have to worry about detrimental environmental factors, such as extreme temperatures, rain, snow, strong winds or air pollution (Heesch et al., 2012). While training indoors, a cyclist can control the temperature and humidity and even simulate different altitudes with hypoxia-inducing procedures.

### Gamification

The gamification of indoor cycling, with feedback loops commonly employed in video games, has lead to a myriad of possibilities for interactive usage that enhances engagement (Beatty, 2013). With the virtual training platform, successful performance is rewarded with special currency, experience points and levels that can be used to make in-game purchases, e.g., bike frames and wheelsets with properties (better aerodynamics or lighter weight) that can improve performance. As has been shown in connection with many exercise tasks (Van Der Kooij et al., 2019; Van Mastrigt et al., 2020), such rewards may motivate users and encourage them to exercise at higher speeds, climb more meters or ride for longer periods to accumulate even greater rewards

In addition, at random points along the course, virtual cycling platforms offer temporary events, called power-ups, that can boost performance, ranging from a reduction in drag to a decrease in the cyclist's body mass, a feature similar to those in many video games.

This can both attenuate the perceived level of exertion, thereby promoting more prolonged and/or intense cycling, and make the experience more versatile and enjoyable (Farrow et al., 2019).

Moreover, the gaming nature of this program may attract new participants by including music and social interactions (e.g., multiplayer options that allow friends to be included or guidance to be received from experienced players), as well as reducing frustration due to poor-quality graphics and overly complex controls and display functions that may evoke motion sickness (Faric et al., 2019).

Finally, virtual forms of training may allow players to engage in more physical activity thereby reducing screen time and self-efficacy (Staiano et al., 2017).

## Weaknesses

### Accuracy

Questionable accuracy has been one of the most obvious weaknesses of Zwift (Whiting, 2018). The many types and models of trainers involved require multiple ways of measuring power output. Some trainers have built-in power meters; others require external devices; and some require speed and cadence sensors which use Zwift's own algorithms to estimate power output. Alternatively, meters on the crank-arm, wheel hub or pedal of the cycle, each with its own level of accuracy, can be used to monitor power.

Zwift applies an algorithm to convert this measured power output to in-game speed. This offers a somewhat crude estimation of actual speed since, as explained in more detail elsewhere, it is based on several factors, including the cyclist's mass, height and choice of bike (Schlange, 2020a).

In this context, aerodynamics, which have a considerable influence on outdoor cycling (Atkinson et al., 2003), are only measured in basic terms of height and mass, with adaptations for specific in-game bicycle and wheel choices. The cyclist's body size and shape are not considered, nor is their riding positions. Cyclists with superior technique and flexibility may be able to assume more aerodynamic positions than others, but this has no impact in-game.

At present, for the devices commonly employed to measure power, manufacturers report a variance in accuracy of ±1–3% (TacX, Wahoo, Elite, 4iiii and Stages). Although this may not be important to a recreational rider, for a competitive cyclist it could well mean the difference between winning and losing. Therefore, for appropriate simulation and interpersonal racing in Zwift, this accuracy must be improved. At present, elite cyclists must verify their Zwift power data with a secondary measuring device, which entails additional expense and technical experience.

The cyclists using “dumb” trainers, with only speed and cadence monitors and no power measurement device, make use of Zwift's alternative algorithms for estimating power (Zwift Power or ZP). Within the racing community these are not considered reliable, and many races exclude riders using these algorithms when reporting results. This could lead to simulated high-level racing becoming an elitist sport.

### Indoor vs. Outdoor Load Metrics

Many recreational and competitive cyclists train both indoors and outdoors over the course of a season. Depending on the technology involved, cyclists may perceive these two types of training differently. In fact, power output and heart rate during cycling outdoors and indoors may differ (Mieras et al., 2014). Thus, internal and external load metrics associated with indoor and outdoor cycling cannot be applied interchangeably.

### Inaccurate Data Entry and “Cyber-Doping”

For the estimated power and actual power algorithms offered by Zwift to function, the user must provide body mass and height to establish an individual drag coefficient for drafting, riding solo, leading groups, or riding up- or downhill. Some cyclists may not know their actual mass and therefore enter incorrect data. False data can affect performance outcomes, since Zwift utilizes watts per kilogram body mass as the main determinant of avatar speed. Moreover, entering an incorrect height would alter the drag coefficient, the second determinant of avatar speed. More concerningly, the cyclist may deliberately enter an incorrect body mass and/or height to improve apparent performance, a practice nicknamed “cyber-doping” and seen by the Zwift community as analogous to real-life doping. There have also been cases of gender swapping, most frequently by men, who then participate in competitions for women only.

So far, these practices have been policed by the community itself, with users flagging suspect performances or requesting verification of power data and/or body mass through external forums. The most common and supposedly robust enforcement involves suspending a cyclist until his/her power data is verified by a secondary power source, although this approach is not always readily available and entails additional cost. Riders can also be suspended until a verified weigh-in video is provided, but this is rare and more questionable, as weighing scales are often poorly calibrated.

Such factors can lead to confusion when reporting results. The results of traditional (non-virtual) elite races are released almost immediately, allowing athletes, teams, and sponsors to celebrate their successes. Zwift's requirements for verification of performances that are suspect could reduce confidence in the results and undermine public perception of the races.

### System Failure

Another weakness of the Zwift system are dropouts, i.e., shorter- or longer-term loss of Bluetooth or Ant+ connectivity between power meters, trainers or computing devices used for simulation. The racing community calls these events “cyber mechanicals,” in analogy to the mechanical failures seen in non-virtual bike races. Dropouts are relatively rare, sometimes only lasting a matter of seconds, but since they may occur at any time, these can still exert a considerable impact on apparent performance, especially during a race. Faulty hardware, problems with software including bugs and/or hosting, and human error (such as not charging devices) can all lead to dropouts. Regardless of the cause, dropouts constitute a risk that sponsors and/or athletes may find unpalatable.

### The Human Component

The very nature of the simulation may reduce the skillful technique and bike handling needed for success in elite non-virtual races. Because of the way it is constructed, sprinting maximally on an ergometer is different to sprinting outdoors. The platform simulates cornering, so there is no need for the user to do so. Furthermore, it is not necessary to understand body positioning while descending or braking and distance management within a group of cyclists. Onscreen avatars and power data make it difficult to determine whether attacks or changes in pace will have the desired effect. Crowds, which can provide emotional support and a sense of gratification when successful, are absent. This may reduce the enthusiasm of both the competitor and sponsors. Furthermore, the overall performance of elite cyclists can be affected by the skills and characteristics of their teammates (e.g., cyclists often try to help the team leader win at the cost of their own chances) (Torgler, 2007). In general, virtual racing may attenuate the intuitive feelings of real-life racing.

## Opportunities

### A New “Normal”

The ongoing Covid-19 pandemic is causing more and more individuals to incorporate virtual platforms into their daily lives. Online exercise and virtual personal training are becoming more common (Thompson, 2021). At the same time, many global sports competitions have been postponed or canceled, opening opportunities for a viable and stable virtual platform to offer alternatives to professional athletic competitions. As long as the availability of live sports events remains limited, the numbers of viewers may increase, and new audiences may be captured.

### New Event Formats, Sponsors, and Teams

Collaboration between event organizers and commercial brands is on the rise, with the first virtual Tour de France in July 2020 (www.letour.fr, 2020). Now teams (some associated with professional teams who compete in Grand Tour races) that focus solely on virtual racing through Zwift are being formed. In addition to the virtual world championships on the Zwift program each year, the three Grand Tours of cycling (Tour de France, Giro d'Italia, Vuelta a España) could also conduct virtual races. The high-profile one-day races could be added as well. In this way elite cyclists could compete year-round with teams and brands exposed to new audiences.

Moreover, the new technology in combination with the pandemic situation presents opportunities for changing the traditional structure of road cycling teams. Cyclists who normally sacrifice personal chances of success by drafting a team leader could race more aggressively, potentially leading to more exciting races and new cycling stars. Furthermore, with shorter races and the ability of each cyclist to prepare nutrition and hydration in advance and keep these close at hand, less time will be lost in this respect and fewer employees assigned to such tasks will be required.

### The Crossover Athlete, Talent Identification, and Coaching

Cyclists who normally specialize in one type of event could try racing in different competitions, e.g., road cyclists could compete in virtual mountain bike races, BMX riders could try gravel racing, etc. Virtual online platforms could also expand to include track cycling disciplines, BMX racing, cyclocross and fixed gear racing. World championship in all-round categories could be offered.

This situation could well-lead to the identification of new talents, with cyclists being particularly successful in virtual disciplines they have not competed in previously. In this manner virtual online cycling platforms could become a testing agency for National Governing Bodies and Olympic Federations. As an example, this is currently operated through an academy, which partners with a professional cycling team to offer a male and female rider a development contract (Norman, 2020), but it could be expanded upon.

Partnering with high-level coaches to provide a greater variety of in-game training plans is another opportunity. With data collected being fed back to the coach and alterations being made where necessary to accommodate training adaptations and responses (Duking et al., 2018, 2020), it is possible that digital online coaching could reduce incidents of overtraining and injury. This could also be accomplished through additional algorithms that automatically adjust the resistance of “smart” trainers when an athlete is training at an intensity that is too high (Duking et al., 2020). Outdoor cycling data could also be added to these algorithms to create a more complete training plan. Such digital coaching framework (Duking et al., 2018) could allow athletes to exercise and train with a quality they may not normally have access to. There are currently external platforms providing this service (e.g., Today's Plan and others), but this approach could be included within the virtual online training platform itself, thereby increasing usage and control.

Finally, virtual platforms provide athletes with physical or cognitive disabilities with opportunities they do not have in the real world. Paralympic disciplines can be included, allowing for increased inclusion and diversity.

### Enhanced Modeling and Simulation

Combining power readings with video data captured from multiple angles could allow for more accurate avatar modeling, thereby increasing the realistic nature of in-game performance. Photographs or video of riders in their preferred riding positions on the bicycle could be beneficial to those with certain body shapes or greater flexibility.

Additionally, virtual online training platforms could simulate weather simulations that a rider might normally encounter, including changes in temperature, wind direction and speed, and rain or snow. This would require cyclists to adapt their tactics to cope with the changes (such as sheltering from head and crosswinds when riding in a group or reducing visibility and avatar responsiveness when riding in rain or snow).

Virtual online platforms may offer opportunities for field-based studies related to both the training and racing aspects of cycling, and the inter-relationship between the two, as all exercises are performed using the same platform and equipment, and may offer the opportunity to recruit many participants.

In this context the so-called “ERG (short for ergometer) mode” allows the resistance of this device to be set automatically. Use of this mode requires a smart bike trainer in combination with either a compatible app or computer that makes it possible to adjust the resistance remotely and maintain constant power output during a workout.

## Integrating aspects of eSPORTS

Virtual athletic platforms will lead to the development of new tactics that could enhance public engagement and excitement. This may explain, at least in part, the surge in popularity of eSports, with tickets for multi-day elite competitions selling out. First events in endurance sport (Ltd, 2020; Triathlon, 2020), a fusion of real-life and virtual triathlon and cycling, immerses fans in a view of the world's best athletes and provides them with actual power, speed and heart rate data collected by Zwift. In addition to attracting new fans, this concept could provide more revenue for athletes, teams, and sponsors.

### In-Game Success and Real-World Advantages

By offering discounts on specific products based on, e.g., the distance cycled, meters climbed, or points accumulated through racing, sponsors could entice users through the gamified feedback loops. To a limited extent, in-game uniforms and unlockable bikes are already offered as rewards for the completion of specific rides or challenges and this could easily be extended to benefits in real life.

This strategy to promote health would allow governments, organizations concerned with public health, and insurance companies to reward users for participation with vouchers or promotional codes.

### Expansion Into New Sports

At present cycling is the leading sport in terms of virtual simulation (through platforms such as Zwift, Real Grand Tours, Rouzy, Sufferfest, Peloton, and TrainerRoad), but the use of analogous simulations for running (Zwift, NordicTrack, Peloton) is expanding. Opportunity exists for expansion into other sports for which reliable indoor training equipment is available, such as rowing and cross-country skiing. In the case of rowing, online comparison of performances has been available for some time, but without any virtual simulation.

## Threats

### Cheating

Cheating remains the foremost threat to Zwift. In the case of some trainers, participants have succeeded in “hacking” and controlling the power-meter remotely (Yeager, 2019). Since some participants have been accused of “cyber-doping,” a Zwift Anti-Doping Agency (“ZADA”) was installed to penalize fiction wattage, misrepresentative metrics and gender swapping (Yeager, 2018).

### Lack of Acceptance

Expansion of virtual competitions will require a certain level of acceptance by existing teams, athletes, coaches, sponsors, and organizers of competitions. If only a few parties accept such virtual competition, it may be viewed as less legitimate and thereby struggle to maintain interest, generate sufficient revenues, and even survive (Akenhead and Nassis, 2016).

In the end, virtual training and competition may come to be a fad (Best, 2006). Especially when the current global pandemic restrictions end and people leave their homes freely to exercise and socialize, they may prefer to return to “real-world” experiences. However, the recent worldwide survey of fitness trends for 2021 indicates clearly that virtual training is not simply a fad (Thompson, 2021).

### Lack of Competition

At present Zwift is the market leader for simulated cycling competition, but without serious competitors it could become an echo chamber of sorts, reducing the drive for innovation and development that might occur if there were competitors of a similar standard.

### Competition or Recreation

Currently, Zwift offers opportunities for competitive and recreational users, but there may come a point in the future when one of these markets is more viable than others. The community aspect drives most daily users, with substantially more people using the platform for training and non-structured riding than racing and competition. Previous research has shown that exergaming supports feelings of competitiveness among those who already identify as competitive and has detrimental effects on those who identify as less competitive (Song et al., 2013). It is possible that the pattern for virtual online platforms is similar, with only those identifying as competitive feeling engaged by the racing aspect of the platform. Racing raises the profile of athletes, brands, and sponsors in a way that recreational use will not, but if user feedback on the recreational component is more positive, then racing may fade from prominence.

### Health Risks

There is some risk that while competing virtually, athletes exercising at-home may push themselves beyond their own safe physical limits and experience an adverse reaction (e.g., injury, nausea, fainting, or injury) in a situation where no supervision or support is available. Cycling indoors without adequate air flow for cooling and sufficient intake of fluids can result in dehydration, thereby imposing additional physiological strain on the cyclist (Ramos-Jimenez et al., 2014).

### Data Security

Finally, the large amounts of data provided by users of virtual programs for training and competition are prone to hacking (Yeager, 2019) and must be protected from inappropriate external access (Spiegel, 2018).

## Summary

**Figure 1:**
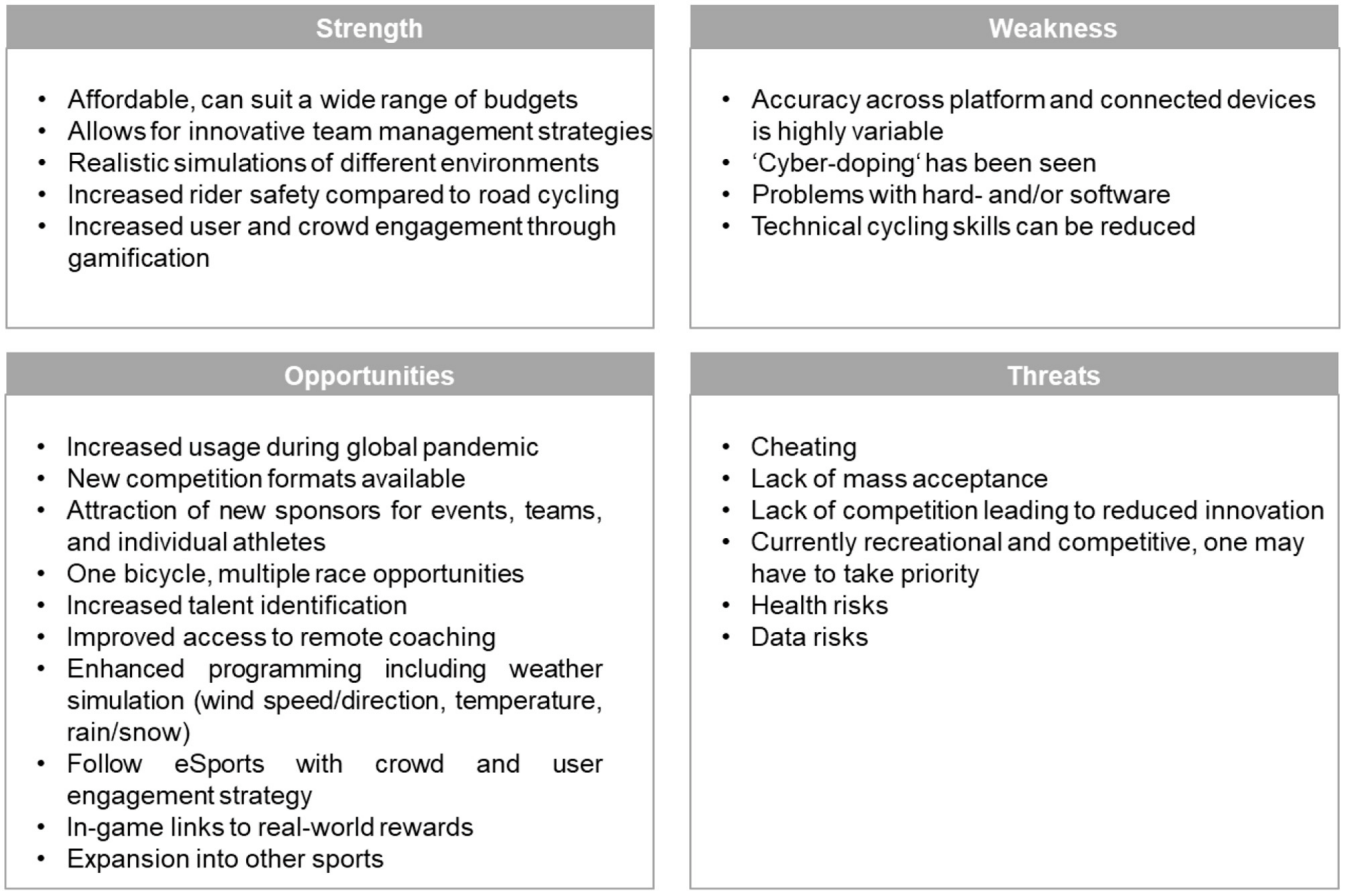
A summary of strengths and weaknesses, as well as opportunities and threats associated with virtual training in cycling.

Virtual training may offer many strengths, opportunities, weaknesses, and threats to cyclists engaging in this new technology, as summarized in Figure 1.

In conclusion, virtual online cycling platforms can build upon its strengths of immersive worlds, innovative drafting mechanics, and versatility by enhancing realism, improving data accuracy, and increasing the strength and reliability of its power-speed algorithms. Opportunities exist for expanding strategic partnerships with major cycling races, brands, and sponsors. User experience can be improved with the addition of video capture and “e-coaching.” Threats are present in the form of cheating, a lack of acceptance and usage by a broader community, health risks and data insecurity.

## Data Availability Statement

The original contributions presented in the study are included in the article/supplementary material, further inquiries can be directed to the corresponding author/s.

## Author Contributions

All authors listed have made a substantial, direct and intellectual contribution to the work, and approved it for publication.

## Conflict of Interest

The authors declare that the research was conducted in the absence of any commercial or financial relationships that could be construed as a potential conflict of interest.
